# Thoracic Saccular Aortic Aneurysm Presenting with Recurrent Laryngeal Nerve Palsy prior to Aneurysm Rupture: A Prodrome of Thoracic Aneurysm Rupture?

**DOI:** 10.1155/2012/367873

**Published:** 2012-04-10

**Authors:** Masafumi Ohki

**Affiliations:** Department of Otolaryngology, Saitama Medical Center, 1981 Kamoda, Kawagoe-shi, Saitama 350-8550, Japan

## Abstract

Left recurrent laryngeal nerve palsy rarely results from cardiac disease. We present 2 cases of left recurrent laryngeal nerve palsy caused by thoracic saccular aortic aneurysms. One patient suffered an aortic aneurysm rupture one month after the advent of hoarseness, necessitating emergency surgery with aortic arch replacement. The other patient underwent elective aortic arch replacement surgery. Both saccular aortic aneurysms protruded downward in the aortopulmonary window to compress the recurrent laryngeal nerves. This is only the 5th case report of the rare occurrence of acute recurrent laryngeal nerve palsy subsequent to saccular aneurysm rupture in the English literature. Recurrent laryngeal nerve palsy does not always indicate imminent aneurysm rupture, but should trigger awareness of a potential rupture in the near future. Left recurrent laryngeal nerve palsy might be a prodrome of aneurysm rupture.

## 1. Introduction

Hoarseness is rarely caused by cardiovascular diseases. Ortner first reported hoarseness due to paralysis of the left recurrent laryngeal nerve caused by a dilated left atrium in mitral stenosis [1]. Recurrent nerve palsy secondary to cardiovascular diseases is called Ortner's syndrome. The primary cardiovascular diseases related to recurrent laryngeal nerve palsy are mitral valve disorders, for example, mitral stenosis, cor pulmonale, pulmonary hypertension, aortopulmonary window, Ebstein's anomaly, aortic aneurysm, aortic dissection, and so on [2, 3]. We experienced 2 rare cases of hoarseness due to left recurrent laryngeal nerve paralysis caused by thoracic saccular aortic aneurysms. One patient manifested acute occurrence of hoarseness and subsequent aneurysm rupture. There are only 4 such case reports of acute recurrent laryngeal nerve palsy with subsequent saccular aneurysm rupture in the English literature [4–7]. We discuss the features of these cases and our experience.

## 2. Case Presentation

### 2.1. Case  1

**Figure 1:**
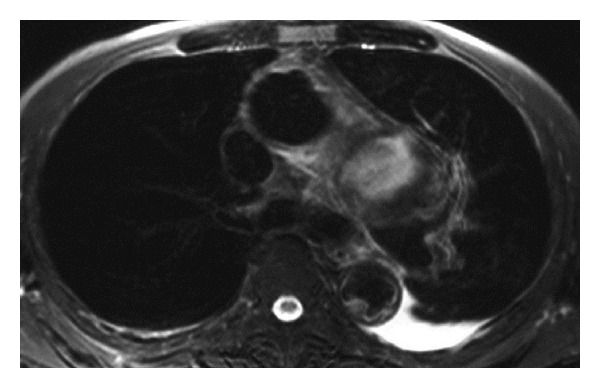
Transverse MRI (T2-weighted image) of the chest revealing a thoracic aortic saccular aneurysm protruding downward in the aortopulmonary window.

**Figure 2:**
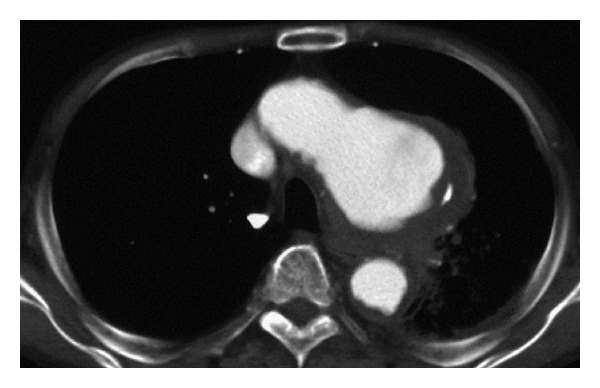
Transverse contrast-enhanced CT of the chest showing an enlarged aortic saccular aneurysm, protruding downward, surrounded by a hematoma in the aortopulmonary window.

A 77-year-old man presented with a 1-month history of hoarseness, sputa, and aspiration. He had no history of severe chest or back pain. His medical history included chronic renal failure, requiring maintenance hemodialysis. Fiberoptic laryngoscopy revealed left vocal cord palsy in the intermediate position. Magnetic resonance imaging (MRI) of the brain and cervix showed normal findings. Chest X-ray showed a widened mediastinum. MRI of the chest revealed a thoracic aortic saccular aneurysm protruding downward in the aortopulmonary window (Figure 1). Ten days later, he presented chest pain and computed tomography (CT) of the chest showed an enlarged aortic saccular aneurysm, protruding downward, surrounded by a hematoma in the aortopulmonary window (Figure 2). He was diagnosed as having a ruptured thoracic aortic arch aneurysm. He underwent urgent surgery for replacement of the ascending aorta and the aortic arch by blood vessel prosthesis. The aorta consisted of a thrombotic saccular aneurysm compressing the main pulmonary arteries in the arch of the aorta and severe atherosclerosis extended from the ascending aorta to the distal aortic arch. Recurrent laryngeal nerve palsy persists, to date, 1 month after surgery.

### 2.2. Case  2

**Figure 3:**
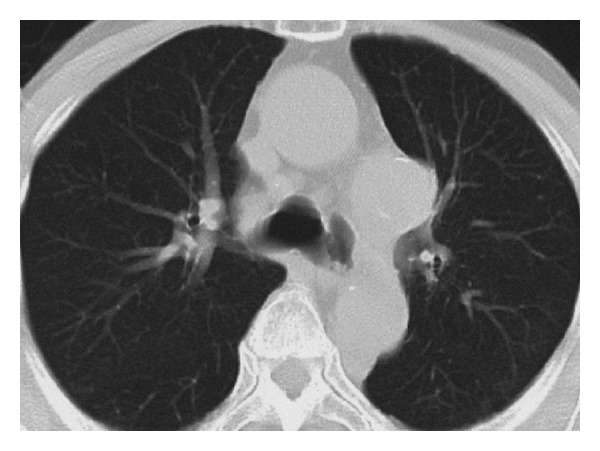
Transverse CT of the chest showing an aortic saccular aneurysm, protruding downward, in the aortopulmonary window.

An 85-year-old female manifested hoarseness for 1 month. She had no history of severe chest or back pain. Fiberoptic laryngoscopy revealed left recurrent laryngeal nerve palsy in the abducted position. Chest X-ray showed a widened mediastinum. CT of the chest revealed a thoracic aortic saccular aneurysm, protruding downward, in the aortopulmonary window (Figure 3). She underwent surgery for replacement of the distal aortic arch by blood vessel prosthesis. The distal arch of the aorta consisted of an atherosclerotic saccular aneurysm, which was 40 mm in diameter, protruding downward and compressing the recurrent laryngeal nerve. The recurrent laryngeal nerve palsy persists, to date, 10 days after surgery.

## 3. Discussion

A true aneurysm involves all 3 layers of the arterial blood vessel wall. Shapes are described as fusiform or saccular type. A fusiform aneurysm balloons out on all sides of the aorta, while a saccular aneurysm bulges or balloons out on one side. Constant pressure from blood, ejected from the heart, continuously presses the already weakened aneurysm wall. An aneurysm gradually increases in size, progressively weakening the aneurysm wall. Aneurysms have a potential risk for rupture or dissection, which can lead directly to death unless urgent surgery is carried out. Even when surgery for aneurysm rupture is performed, the fatality rate is high. Recurrent nerve palsy due to thoracic aortic aneurysm is an unusual complication. Thoracic aortic aneurysm is the cause of recurrent nerve palsy in 5% of cases [4]. The mechanism of left recurrent laryngeal nerve palsy has not as yet been clarified. However, it is attributed to compression of the left recurrent laryngeal nerve hooking around the ligamentum arteriosum between the pulmonary artery and aorta via aneurysm enlargement [3]. In our cases, saccular aneurysms protruded into the space between the pulmonary artery and aortic arch, apparently compressing the recurrent nerve. The advent of recurrent laryngeal nerve palsy in patients with cardiovascular diseases requires prompt detailed examination because it suggests possible aneurysm dilation. Aortic aneurysms are life threatening in cases with rupture. When recurrent nerve palsy occurs, is it a warning sign of aneurysm rupture? In case  1, a thoracic aneurysm ruptured 1 month after the advent of hoarseness. Some reports described aneurysms as rupturing 0 to 3 days after the onset of recurrent laryngeal nerve palsy [4–6], whereas aneurysm rupture could be avoided if recurrent laryngeal nerve palsy prompted elective surgery 1 month to 1 year after its onset [4, 7–11]. Chan reported that death occurred after severe chest pain and subsequent hypotension 1 year after manifestation of recurrent laryngeal nerve palsy in a patient with an aneurysm [12]. Texido described 8 patients with hoarseness due to an aneurysm and one suffered a rupture 1 day after the onset of hoarseness [4]. Recurrent laryngeal nerve palsy does not always mean imminent aneurysm rupture. However, rupture can be imminent. We may regard recurrent nerve palsy as a prodrome of aneurysm rupture. Surgical treatments are mainly of 2 types, that is, conventional open chest surgery and endovascular surgery. Patients may recover from recurrent laryngeal nerve palsy 6 to 12 months after both types of surgery, but this is not always the case [8, 9, 13–15].

## 4. Conclusion

Left recurrent nerve palsy can arise from a thoracic saccular aortic aneurysm. Thoracic saccular aortic aneurysm is an important factor in making the differential diagnosis of recurrent nerve palsy. It might be a prodrome of aneurysm rupture necessitating emergent management.
